# Virtual Touch IQ elastography reduces unnecessary breast biopsies by applying quantitative “rule-in” and “rule-out” threshold values

**DOI:** 10.1038/s41598-018-22065-7

**Published:** 2018-02-26

**Authors:** Panagiotis Kapetas, Paola Clauser, Ramona Woitek, Katja Pinker, Maria Bernathova, Thomas H. Helbich, Pascal A. Baltzer

**Affiliations:** 1 Department of Biomedical Imaging and Image-guided Therapy, Medical University of Vienna, Waehringer Guertel 18-20, 1090 Vienna, Austria; 2 Department of Radiology, University of Cambridge, Cambridge Biomedical Campus, CB2 0QQ Cambridge, UK; 3 Memorial Sloan-Kettering Cancer Center, Molecular Imaging and Therapy Service, 301 E 55th St, 10022 New York, NY USA

## Abstract

Our purpose was to evaluate Virtual Touch IQ (VTIQ) elastography and identify quantitative “rule-in” and “rule-out” thresholds for the probability of malignancy, which can help avoid unnecessary breast biopsies. 189 patients with 196 sonographically evident lesions were included in this retrospective, IRB-approved study. Quantitative VTIQ images of each lesion measuring the respective maximum Shear Wave Velocity (SWV) were obtained. Paired and unpaired, non-parametric statistics were applied for comparisons as appropriate. ROC-curve analysis was used to analyse the diagnostic performance of VTIQ and to specify “rule-in” and “rule-out” thresholds for the probability of malignancy. The standard of reference was either histopathology or follow-up stability for >24 months. 84 lesions were malignant and 112 benign. Median SWV of benign lesions was significantly lower than that of malignant lesions (p < 0.001). The application of a “rule-out” threshold of 1.9 m/s lead to a sensitivity of >98% with a concomitant significant (p = 0.032) reduction in false positive cases of almost 15%, whereas a “rule-in” threshold of 6.5 m/s suggested a probability of malignancy of >95%. In conclusion, VTIQ elastography accurately differentiates malignant from benign breast lesions. The application of quantitative “rule-in” and “rule-out” thresholds is feasible and allows reduction of unnecessary benign breast biopsies by almost 15%.

## Introduction

Ultrasound (US) of the breast is a valuable adjunct to mammography in the characterization of breast lesions. Using established diagnostic criteria, as described in the Breast Imaging Reporting and Data System (BI-RADS) lexicon^1^, US shows a high sensitivity at a variable but in general moderate specificity (6.4–83%)^2–6^.

The need to reduce the false-positive US findings has resulted in research efforts adding further US techniques for lesion evaluation before biopsy^2,7^. Elastography, one of these new promising techniques, evaluates the mechanical properties of tissue. Several approaches have been investigated, such as strain elastography, Shear Wave Elastography (SWE) and Acoustic Radiation Force Impulse (ARFI). Recently a new reconstruction algorithm for ARFI imaging has been developed, namely Virtual Touch IQ (VTIQ)^8^. In contrast to previous approaches, VTIQ enables a simultaneous qualitative and quantitative evaluation of tissue stiffness, measuring Shear Wave Velocities (SWV)^8^. Due to both the new algorithm used as well as the smaller ROI size (2 × 2 mm compared to 5 × 5 mm in previous versions), VTIQ has a higher rate of successful measurements as compared to previous versions of ARFI elastography^8^.

Recent studies have shown VTIQ to accurately differentiate between benign and malignant breast lesions, improving the specificity (from 38.7–47.2% to 91.5–94.8% in one series)^9^ and positive predictive value (PPV) of B-mode breast US (from 76% of B-mode US to 81.4%, when applied to BI-RADS 4 lesions in another series)^10^. Intralesional calcifications as well as a high degree of fibroblastic proliferation or stromal hyalinization have been recognized as reasons, that may lead to false positive results, rendering high stiffness measurements in benign lesions^9,11^. However, to our knowledge, the possibility to provide clinically useful, quantitative SWV threshold values that would allow us to “rule-in” or “rule-out” malignancy and evaluate their effect on the number of biopsies performed has not been proposed yet. Such criteria however are mandatory for a clinically useful diagnostic test as they provide the opportunity for unambiguous management decisions such as to perform a biopsy or not^12^. Moreover, the introduction of such threshold values may facilitate a more structured integration of elastography information into the diagnostic process.

Therefore, our aim was to evaluate VTIQ elastography and identify quantitative “rule-in” and “rule-out” threshold values for the probability of malignancy, which can help avoid unnecessary breast biopsies.

## Methods

### Patients

**Figure 1:**
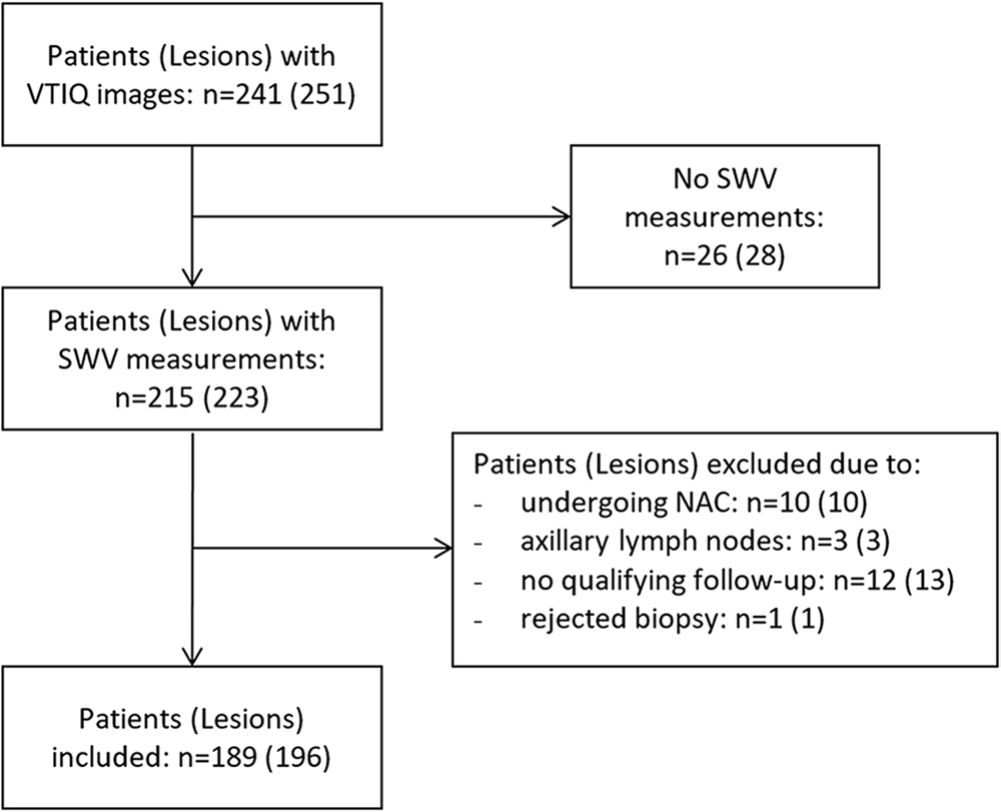
Flowchart showing the patients and lesions included to and excluded from our retrospective study. Number of lesions is shown in parentheses. VTIQ: Virtual Touch IQ, SWV: Shear Wave Velocity, NAC: neoadjuvant chemotherapy.

Institutional Review Board approval was obtained for this retrospective, cross-sectional study by the Ethics Committee of the Medical University of Vienna. Due to the retrospective nature of the study, the requirement for an informed consent by the patients was waived. The study was conducted in accordance with the Declaration of Helsinki. All diagnostic US examinations of the breast, which included ARFI imaging with VTIQ (Siemens Medical Solutions Inc., Mountain View, CA, USA), performed at our institution between June 2013 and June 2015, were retrospectively reviewed by searching the local PACS database. Overall 241 patients with 251 breast lesions were identified. In order to be eligible for inclusion in the study, an examination had to meet the following criteria: a breast lesion visible at B-mode US with at least 5 mm in size (BI-RADS 2–5); no previous biopsy or neoadjuvant chemotherapy; colour coded VTIQ image of the lesion with quantitative measurement of its SWV; histologic confirmation of the lesions, or, in case of a benign lesion (BI-RADS 2), stability for at least 24 months of follow-up. Finally 189 patients with 196 sonographically evident lesions fulfilled the above criteria and were included in the study. The flowchart in Fig. 1 shows the study inclusions and exclusions.

### Data acquisition

All examinations were performed on a Siemens Acuson S3000 US device (Siemens Medical Solutions Inc., Mountain View, CA, USA) with the same software version throughout the time period examined. B-mode images were acquired with a linear 18L6 HD transducer, which was then changed to a different linear 9L4 transducer for VTIQ imaging. No “split-screen” mode was available to us at that time, therefore the B-mode and VTIQ images were acquired separately. On the color-coded VTIQ image of the lesion, the examiner placed a 2 × 2 mm quantification ROI at the stiffest area of the lesion (as appreciated on the color-coded map), in order to measure the SWV. In cases of more than one measurements per lesion, only the one with the highest velocity was included in our analysis, provided that it was obtained from an area of high imaging quality in the quality map. In cases when the system could not achieve a valid measurement and only indicated “High” (that is higher than the maximum measurable velocity of 10 m/s) it was replaced by a value of 10 m/s for the statistical analysis^13,14^.

By consensus, all VTIQ images in our department are obtained by applying minimal compression; that is the least possible compression, in order to achieve a reasonable, artifact-free image. A high degree of precompression when performing SWV measurements has been demonstrated to lead to artificial tissue stiffening and thus measurement of higher velocities^15^.

### Histopathological examination

140 patients received an US-guided biopsy, using either a 14 G core-biopsy needle or a 9 G vacuum-assisted needle. The needle choice depended on the sonographic characteristics of the lesion and was met by the physician who performed the examination. In cases when the biopsy result was that of a lesion with uncertain malignant potential, the final, post-surgical histopathology results were used for our analysis. In all other cases, the results of the histopathological analysis of the biopsy specimen were used as the reference standard.

### Statistical analysis

Statistical calculations were performed using the software SPSS 20 (IBM Corp, NY, USA) and MedCalc 12.5.0.0 (Mariakerke, Belgium).

Descriptive statistics were used to calculate medians and interquartile ranges. The Mann- Whitney *U* test was used to compare the velocities of benign and malignant lesions. Z test was used for proportions, in order to evaluate the significance of the decrease in the number of necessary biopsies. Receiver operating characteristics (ROC) curve analysis was used to evaluate the diagnostic performance of VTIQ.

In addition, the ROC curve was analysed regarding possible “rule-in” and “rule-out” threshold values for the probability of malignancy. To do this, the diagnostic SWV threshold to differentiate between benign and malignant lesions was varied. At appropriate cut-off values the sensitivity, specificity, positive and negative predictive values were calculated^16^. In accordance with BI-RADS^1^, a sensitivity of at least 98% was considered a requirement to downgrade a lesion (“rule-out” criterion) while a specificity of at least 95% was considered a “rule-in” criterion for breast cancer^1^. A p value of less than 0.05 was considered statistically significant.

## Results

### Lesion characteristics

84 lesions were malignant and 112 benign (56 biopsy proven and 56 with a follow-up stability over at least 24 months). The median lesion size was 14 mm (range 5–54 mm). A summary of the histopathology of all lesions and the corresponding median SWV is shown in Table 1. The detailed histopathology of all included lesions and their corresponding SWV can be found in the Supplementary Table S1.

**Table 1:** Summary of the histopathology and shear wave velocities (SWV) obtained by VTIQ elastography from all lesions. Note: *50% of the benign lesions showed stability over a 2 year follow-up. Q1 = first quartile, Q3 = third quartile. Velocities are given in m/s.

Diagnosis	N	Median SWV (Q1; Q3)
**Malignant (n = 84)**		**5.57 (3.58; 8.73)**
Invasive ductal carcinoma	67	5.64 (3.68; 9.07)
Invasive lobular carcinoma	7	6.17 (4.47; 6.82)
Ductal carcinoma *in situ*	5	3.59 (2.43; 8.56)
Mucinous Carcinoma	3	6.83 (6.14; 7.08)
Medullary Carcinoma	1	2.68 (n/a; n/a)
Angiosarcoma	1	3.43 (n/a; n/a)
Benign (n = 112)*		**2.52 (2.05; 3.21)**
Histopathologically verified	56	2.69 (2.12; 3.56)
Fibroadenoma	13	3.21 (2.75; 4.89)
Fibroadenomatoid hyperplasia	8	2.85 (2.55; 3.32)
Inflammatory changes	7	2.36 (1.97; 3.95)
Papilloma	5	3.04 (2.81; 3.56)
Others	23	2.27 (1.90; 2.92)
Follow Up stability	56	2.46 (1.91; 2.87)

**Figure 2:**
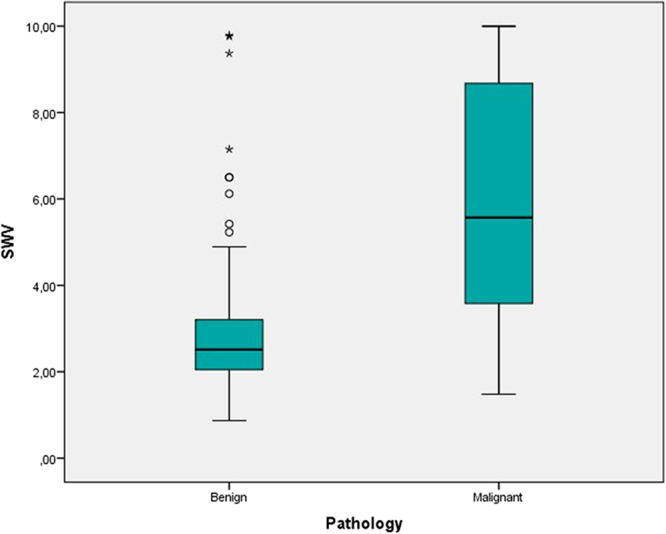
Boxplot, showing the differences between Shear Wave Velocities (SWV) obtained by VTIQ elastography from benign and malignant lesions (p < 0.001).

Median SWV values were 2.52 m/s (Q1 = 2.05 m/s, Q3 = 3.21 m/s) for benign and 5.57 m/s (Q1 = 3.58 m/s, Q3 = 8.73 m/s) for malignant lesions (Fig. 2). The difference in SWV between benign and malignant lesions was statistically significant (p < 0.001).

Out of all 196 SWV measurements, only 2 returned an “invalid” value of X.XX m/s. Both lesions were altogether very hard, as appreciated on the color-coded map and presented a malignant histology.

### “Rule-in” and “rule-out” threshold values

**Figure 3:**
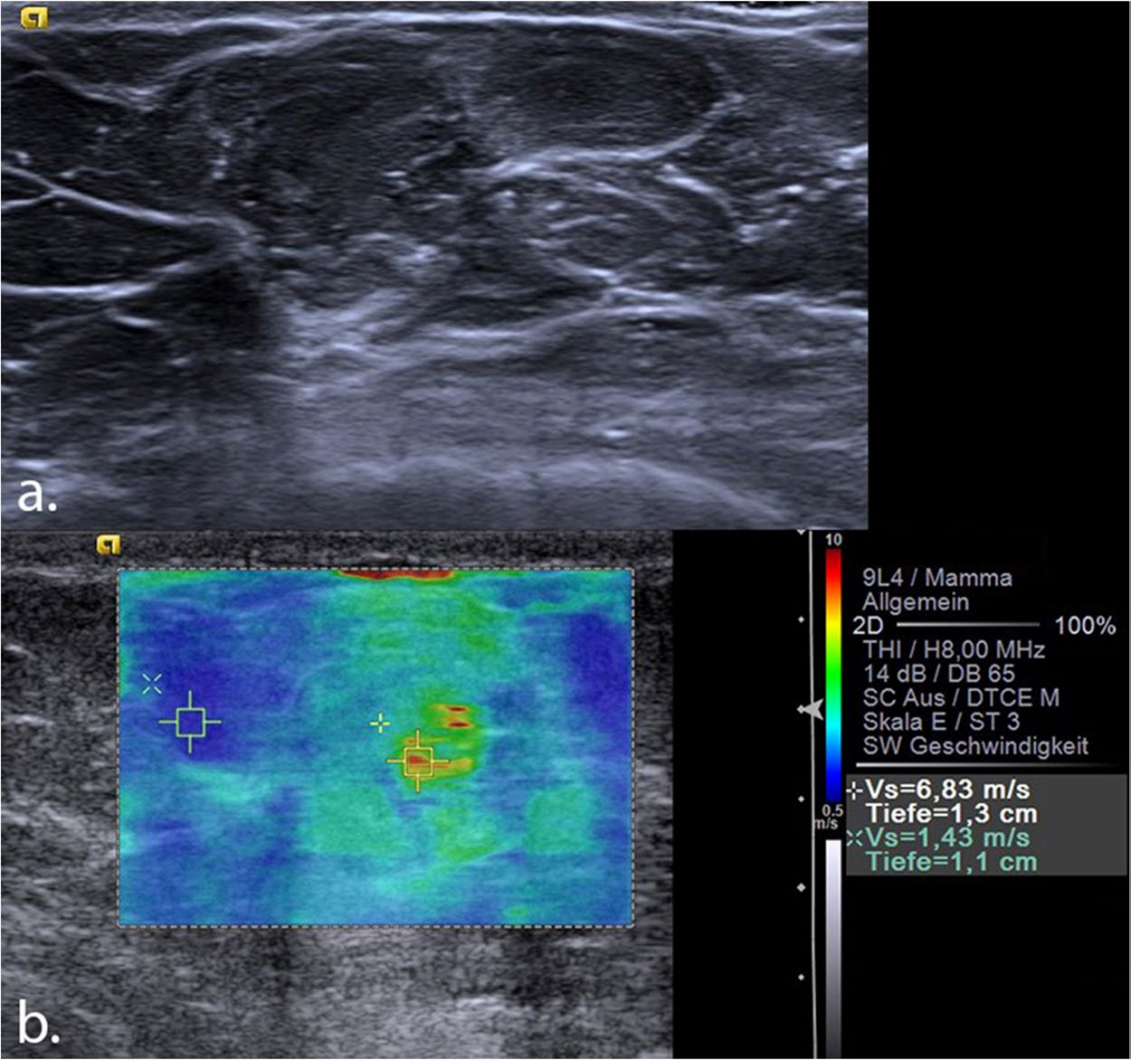
(**a**) Mucinous carcinoma seen in B-mode ultrasound as a 14 mm isoechoic, partially lobulated lesion with posterior enhancement, which was classified as BI-RADS 4b. (**b**) VTIQ elastography shows an overall hard lesion (scale adjusted to 10 m/s) with a maximum shear wave velocity of 6.83 m/s (above the “rule-in” threshold), indicating a BI-RADS 5 tumour. A further ROI measures the velocity inside fatty tissue (1.43 m/s). VTIQ: Virtual Touch IQ, ROI: region of interest.

**Figure 4:**
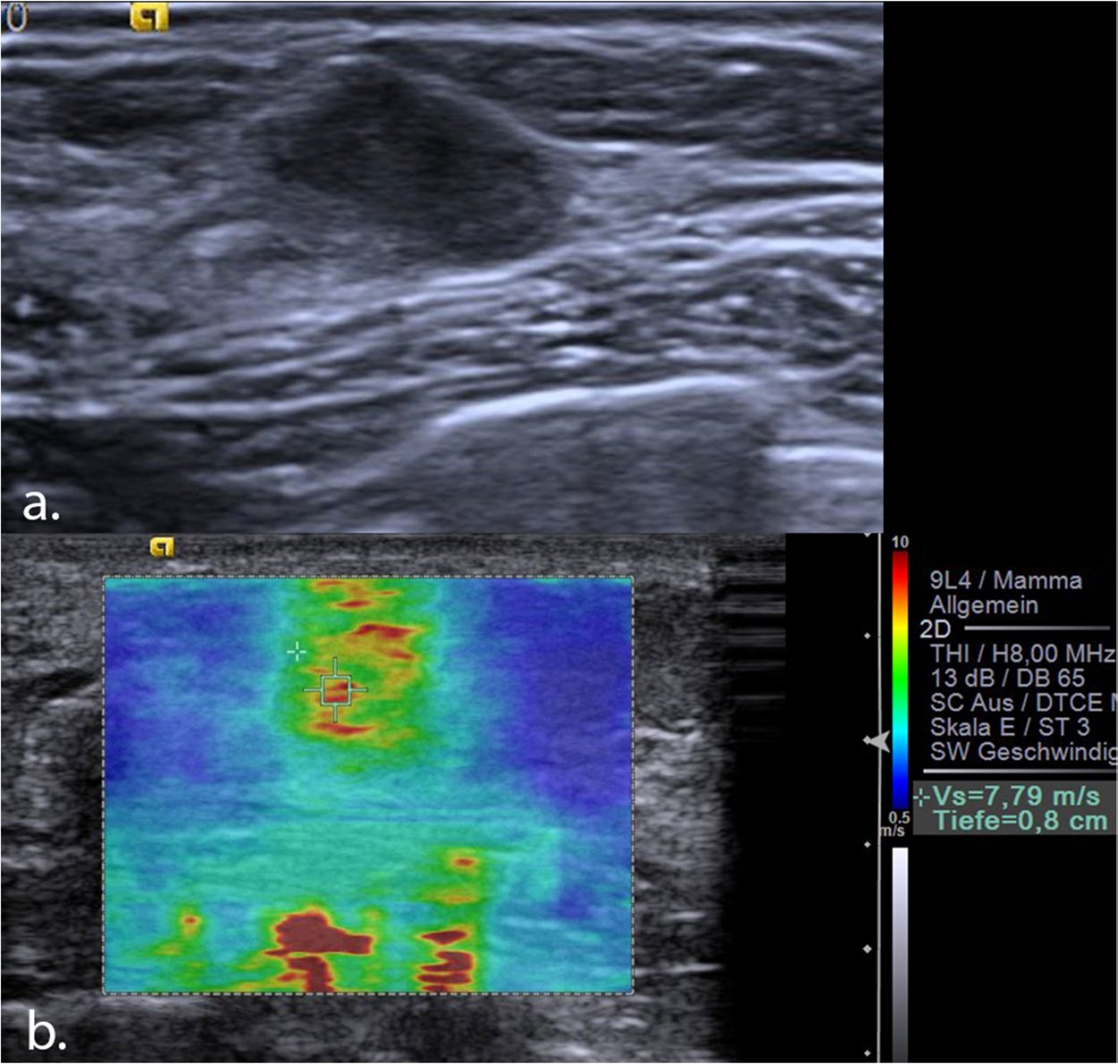
(**a**) Invasive ductal carcinoma Grade 1 seen in B-mode ultrasound as an 8 mm, hypoechoic, circumscribed lesion, which was initially classified as BI-RADS 3. (**b**) VTIQ elastography shows a very hard lesion (scale adjusted to 10 m/s) with a maximum shear wave velocity of 7.79 m/s (above the “rule-in” threshold), indicating a BI-RADS 5 tumour. VTIQ: Virtual Touch IQ, ROI: region of interest.

By varying the SWV cut-off value, “rule-in” and “rule-out” thresholds were specified. A high SWV cut-off value of 6.5 m/s suggested a probability of malignancy of >95% (Figs 3 and 4), leading to six false positive results (two cases of calcified fat necrosis, two abscesses and two fibroadenomas). On the contrary, a low cut-off value of 1.9 m/s led to a sensitivity of almost 99%, with one false negative result (one case of a 7 mm Grade 1 DCIS). Details of the threshold values and their diagnostic parameters are shown in Table 2.

**Table 2:** Different, VTIQ elastography obtained, shear wave velocity (SWV) cut-off values and their resulting diagnostic parameters. Velocities are given in m/s. 95% Confidence intervals are given in parenthesis.

SWV cut-off	Sensitivity	Specificity	PPV	NPV
>1.90	98.8% (93.5–99.8%)	19.6% (12.7–28.2%)	48.0%	95.7%
>3.23	85.7% (76.4–92.4%)	76.8% (67.9–84.2%)	73.5%	87.8%
>6.50	38.1% (27.7–49.3%)	96.4% (91.1–99.0%)	88.9%	67.5%

### Effect of the “rule-out” threshold value on the number of performed biopsies

**Figure 5:**
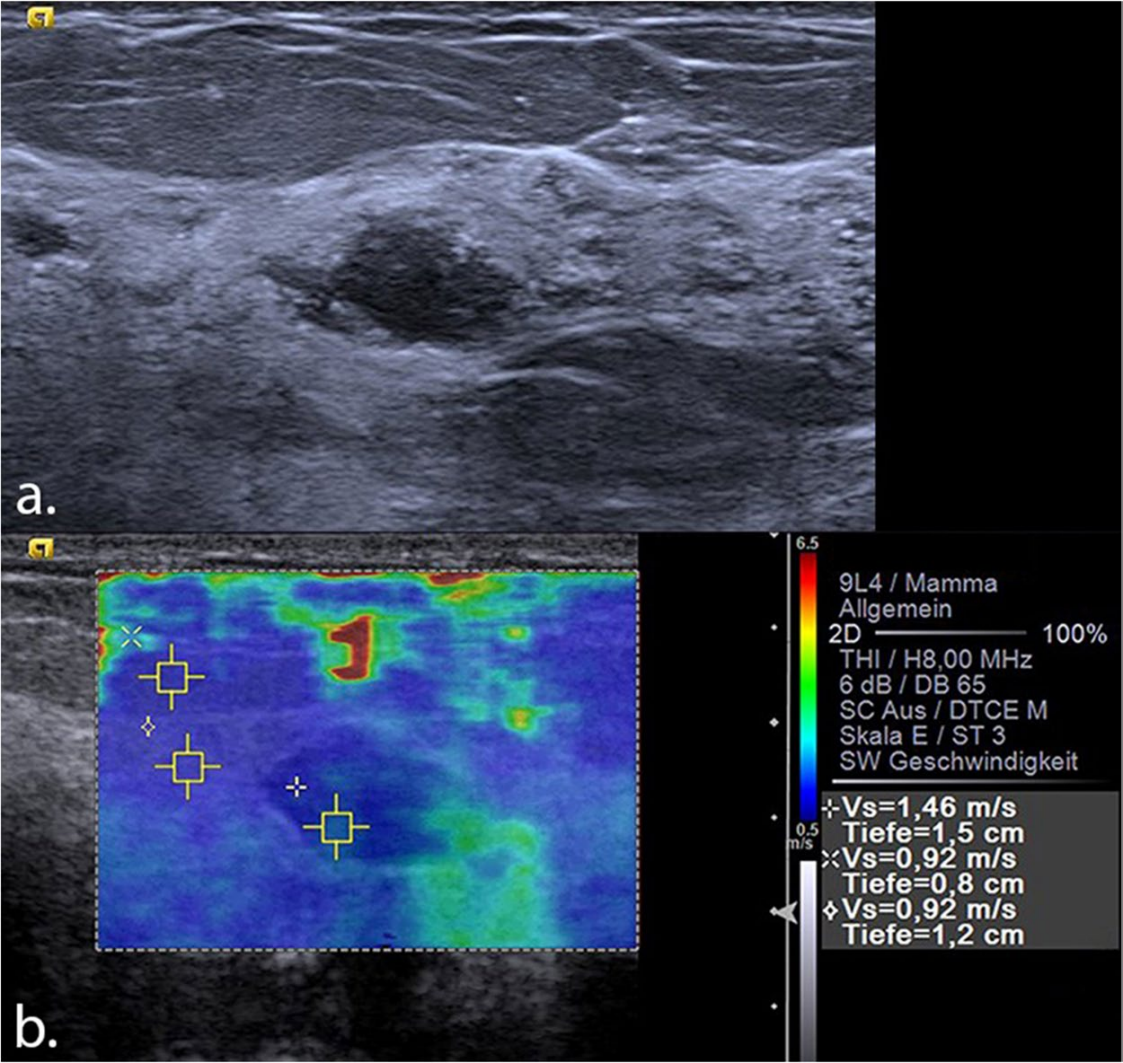
(**a**) Histologically verified sclerosing adenosis seen in B-mode ultrasound as a 12 mm hypoechoic, partially non-circumscribed lesion, which was classified as BI-RADS 4a. (**b**) VTIQ elastography shows a very soft lesion with a maximum shear wave velocity of 1.46 m/s (well below the “rule-out” threshold), thus indicating that it can be safely classified as a BI-RADS 3. Further ROIs measure the velocity inside fatty tissue (0.92 m/s) and breast parenchyma (0.92 m/s). VTIQ: Virtual Touch IQ, ROI: region of interest.

**Figure 6:**
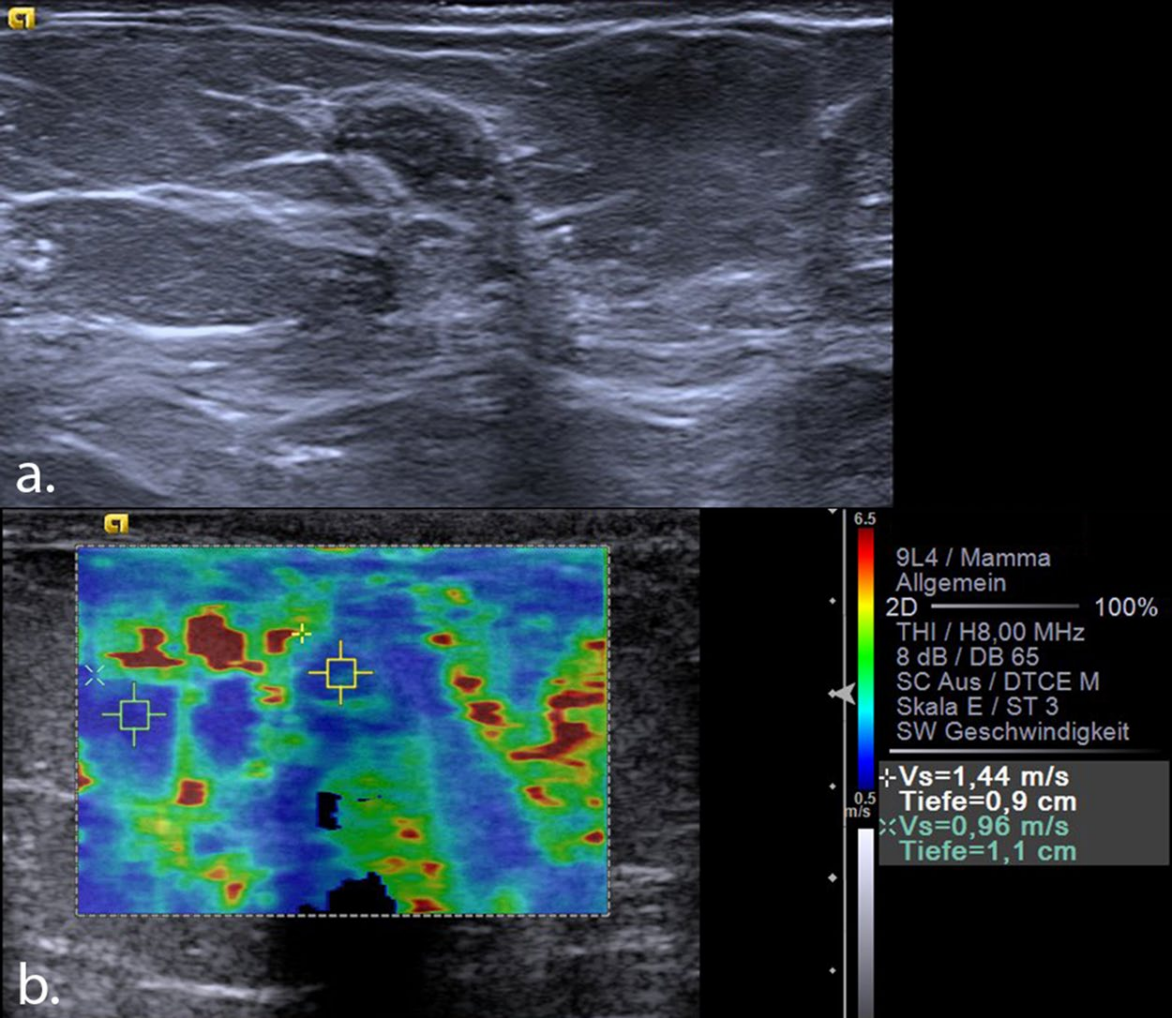
(**a**) Histologically verified area of adenosis seen in B-mode ultrasound as an 11 mm hypoechoic, partially angular lesion, which was classified as BI-RADS 4a. (**b**) VTIQ elastography demonstrates a very soft lesion with a maximum shear wave velocity of 1.44 m/s, suggestive of a benign lesion. A further ROI measures the velocity inside fatty tissue (0.96 m/s). VTIQ: Virtual Touch IQ, ROI: region of interest.

56 lesions that had been classified as BI-RADS 4 by routine B-mode US underwent a biopsy and proved to be benign. By applying the “rule-out” threshold value of 1.9 m/s, 8 out of 56 biopsied benign lesions could have been identified as such and thus the corresponding US-guided biopsies could have been avoided, leading to a statistically significant decrease of 14.3% in the number of benign biopsies (p = 0.032, CI: 23.3–49.6%) (Figs 5 and 6). The histology of the 8 lesions, correctly identified by VTIQ as benign is shown in Table 3.

**Table 3:** Histopathology and corresponding, VTIQ elastography obtained, shear wave velocities (SWV) of the 8 lesions, for which biopsy could have been avoided. Velocities are given in m/s.

N	Histopathology	SWV
1	Fat necrosis	1.81
2	Sclerosing adenosis	1.46
3	Capillary haemangioma	1.42
4	Periductal mastitis	1.86
5	Inflamed cyst	0.87
6	Adenosis	1.44
7	Lipoma	1.18
8	Breast parenchyma with fibrosis	1.00

## Discussion

The results of our study show that a new reconstruction algorithm for ARFI elastography, namely VTIQ, allows substantial differentiation of benign from malignant breast lesions. In addition, “rule-in” and “rule-out” criteria for malignancy, allowing objective and quantitative diagnoses, were identified and led to an almost 15% decrease of unnecessary benign biopsies, while, at the same time, offering a more confident diagnosis of less malignant-looking lesions.

An ideal diagnostic test can aid in ruling-in or ruling-out malignancy. In our study, by applying a SWV cut-off value of 6.5 m/s, the probability of malignancy of a lesion was shown to be higher than 95%, in concordance with the BI-RADS category 5 (highly suggestive of malignancy). Thus, it is reasonable to upgrade a lesion that looks otherwise less suspicious in B-mode US, if it shows a SWV higher than the aforementioned cut-off value, due to the high PPV of the “rule-in” cut-off. In case of a benign histopathology on biopsy in lesions presenting with “rule-in” SWV values, concordance must be reviewed and either re-biopsy or short-term surveillance should be initiated. The application of this cut-off value would lead to only six false positive results in our series: two cases of calcified fat necrosis (readily identified in mammography), two abscesses (with correlating clinical symptoms) and two fibroadenomas. As has been already mentioned, usual reasons for false positive results in VTIQ are intratumoral calcifications and a high degree of fibroblastic proliferation or stromal hyalinization^9,11^. Moreover, these false positive cases underline the significance of taking into account the clinical and previous mammographical findings for the interpretation of breast US in general and VTIQ specifically.

On the other hand, a SWV threshold of 1.9 m/s leads to a sensitivity of almost 99%, with a subsequent false negative rate of less than 2% and a negative predictive value (NPV) of >95%. This allows a downgrade of otherwise suspicious (i.e. BI-RADS 4) lesions to the BI-RADS 3 category, without missing any substantial number of cancers, while at the same time the number of false positive cases in B-mode US and, more significantly, the number of unnecessary biopsies can be reduced by almost 15%. In fact, the only case that would have been missed in our study was a 7 mm Grade 1 DCIS, most likely due to the lack of any considerable desmoplastic reaction in the surrounding parenchyma. No cases of invasive cancers or intermediate or high grade DCIS presented a SWV of less than 1.9 m/s in our series. This finding might be of special importance in an era when overdiagnosis, that is the discovery of breast cancer, which is not biologically significant, poses a central debate^17^.

Several SWV threshold values for the differentiation between benign and malignant breast lesions have been proposed in literature, focusing on the best statistical discriminator leading to the possibly highest sensitivity while limiting false positive findings. In concordance to previous work from our group^11^, ROC curve analysis in our present study suggested an optimal threshold of 3.23 m/s with a sensitivity of 85.7% (95% CI: 76.4–92.4%) and a specificity of 76.8% (95% CI: 67.9–84.2%). Other studies have proposed similar (3.31–3.68 m/s)^10,18^ or quite divergent (5.18–6.593 m/s)^19,20^ threshold values, with sensitivities ranging between 80.4% and 98% and specificities between 62.6% and 96.5%. However, at these threshold values there is usually a considerable overlap between benign and malignant lesions, which precludes unequivocal diagnosis and does not allow for a lesion to be classified as either benign or malignant with certainty. On the other hand, the application of “rule-in” and “rule-out” criteria for malignancy can accurately differentiate benign from malignant breast lesions and offer a structured combination of elastography findings with lesion morphology assessed with the B-mode BI-RADS descriptors.

It has to be stressed, that these cut-off values have been calculated based on VTIQ measurements with a very low precompression. There is an unavoidable degree of inter-examiner variability, although ARFI imaging (similarly to SWE) shows a high reproducibility among different examiners^11,21^. Moreover, a recent study has shown that there is variance between SWVs measured with different devices of different vendors, which means that our results should only be used with caution in examinations performed with different US apparatus^22^. It seems rational to calibrate each system regarding reproducibility and SWV thresholds in order to avoid biased measurements.

US-guided biopsy is readily available throughout most breast centres, has a high diagnostic accuracy and is generally considered a safe way of tissue acquisition^23–26^, when appropriate morphologic criteria are met^27,28^. However, like any other interventional technique, US-guided biopsy is associate with complications, though minor^25^. Moreover, both the suggestion to perform a biopsy as well as the time elapsing between the biopsy performance and the delivery of the results represent a psychological burden for the patient. Finally, substantial costs arise from interventional procedures for the healthcare systems throughout the world^29^, although image-guided biopsies are associated with lower expenditures than open surgical procedures. Thus any imaging modality that leads to a reduction of unnecessary breast biopsies has to be appreciated.

An attempt to establish threshold values that effectively “rule-in” or “rule-out” malignancy in order to avoid unnecessary breast biopsies has already been made for different MRI modalities. Woitek *et al*. used morphological lesion characteristics and a classification algorithm and could demonstrate a possible reduction in benign breast biopsies by more than 25%^30^. With a similar approach, Spick *et al*. showed that the application of “rule-in” and “rule-out” threshold values in quantitative diffusion weighted imaging has the potential to reduce the number of unnecessary breast biopsies by almost 35%^16^. To our knowledge, this is the first attempt to introduce quantitative “rule-in” and “rule-out” thresholds for the probability of malignancy in sonographic SWE and more particularly in ARFI imaging with VTIQ. Although the percentage decrease in unnecessary benign breast biopsies in our study (almost 15%) was lower than the one achieved in the two aforementioned studies, one should consider the fact, that the large majority of image-guided biopsies performed worldwide is under US guidance, as compared to the much more limited number of MRI-guided breast biopsies. It may thus be assumed that reducing the rate of unnecessary US-guided biopsies by only a small amount does have a much higher impact regarding the absolute number and costs of avoided biopsies.

The main limitation of our study is its retrospective nature, which allows for a possible selection bias, since the cases included were found by searching the local PACS database. It has to be noted though, that VTIQ is routinely used in our department. Moreover, a direct comparison of the probability of malignancy as appreciated by the SWV (according to the calculated cut-off values) with the actually, clinically assigned BI-RADS category of each lesion was not performed- although our study data let assume an improved specificity using “rule-out” SWV criteria, this has to be tested in an independent validation study, with more examiners and a larger number of participants, preferably in a multicentric setting.

In conclusion, our study demonstrates that VTIQ elastography aids in the accurate differentiation of malignant from benign breast lesions and the application of quantitative “rule-in” and “rule-out” threshold values is feasible and allows reduction of unnecessary benign breast biopsies by almost 15%.

### Data availability statement

The datasets generated during and/or analysed during the current study are available from the corresponding author on reasonable request.

## Electronic supplementary material

Supplementary table S1
