# Supplementary material for: Virtual Touch IQ elastography reduces unnecessary breast biopsies by applying quantitative “rule-in” and “rule-out” threshold values
Source: Sci Rep. 2018 Feb 26;8:3583. doi: 10.1038/s41598-018-22065-7 (PMC5827686; doi:10.1038/s41598-018-22065-7)
Supplement: Supplementary file 1 — Supplementary table S1 [file 41598_2018_22065_MOESM1_ESM.docx]

**Virtual Touch IQ elastography reduces unnecessary breast biopsies by applying quantitative “rule-in” and “rule-out” threshold values**

Panagiotis Kapetas^1^, Paola Clauser^1^, Ramona Woitek^1,2^, Katja Pinker^1,3^, Maria Bernathova^1^, Thomas H. Helbich^1*^, Pascal A. Baltzer^1^

^1^ Department of Biomedical Imaging and Image-guided Therapy, Medical University of Vienna, Waehringer Guertel 18-20, 1090, Vienna, Austria

^2^ Department of Radiology, University of Cambridge, Cambridge Biomedical Campus, CB2 0QQ, Cambridge, UK

^3^ Memorial Sloan-Kettering Cancer Center, Molecular Imaging and Therapy Service, 301 E 55th St, 10022, New York, NY, USA

^*^ Correspondence to: Thomas H. Helbich, Department of Biomedical Imaging and Image-guided Therapy, Medical University of Vienna, Waehringer Guertel 18-20, 1090, Vienna, Austria. Email: thomas.helbich@meduniwien.ac.at

| Patient number | Breast | Clock position | Size (mm) | SWV (m/s) | Histopathological verification | Histology |
| --- | --- | --- | --- | --- | --- | --- |
| 1 | R | 9 | 50 | 9.95 | US-guided biopsy | IDC Grade3 |
| 2 | L | 10 | 15 | 4.95 | US-guided biopsy | IDC Grade1 |
| 3 | R | 9 | 9 | 3.31 | US-guided biopsy | IDC Grade2 |
| 4 | L | 4 | 9 | 3.43 | US-guided biopsy | IDC Grade2 |
| 5 | R | 9 | 11 | 3.25 | US-guided biopsy | IDC Grade2 |
| 6 | R | 7 | 11 | 2.23 | US-guided biopsy | IDC Grade2 |
| 7 | R | 10 | 25 | 9.96 | US-guided biopsy | ILC Grade2 |
| 8 | L | 3 | 26 | 9.91 | US-guided biopsy | IDC Grade1 |
| 9 | L | 2 | 15 | 5.45 | US-guided biopsy | Mucinous carcinoma Grade1 |
| 10 | R | 1 | 5 | 4.45 | US-guided biopsy | IDC Grade3 |
| 11 | R | 8 | 25 | High (10.00) | US-guided biopsy | IDC Grade3 |
| 12 | R | axillary tail | 45 | 8.45 | US-guided biopsy | IDC Grade3 |
| 13 | L | 2 | 8 | 2.53 | US-guided biopsy | IDC Grade2 |
| 14 | L | 6 | 10 | 3.37 | US-guided biopsy | IDC Grade3 |
| 15 | L | 2 | 12 | 3.94 | US-guided biopsy | ILC Grade2 |
| 16 | L | 10 | 17 | 9.80 | US-guided biopsy | IDC Grade3 |
| 17 | L | 8 | 25 | 9.57 | US-guided biopsy | IDC Grade2 |
| 18 | L | 12 | 12 | 4.85 | US-guided biopsy | IDC Grade2 |
| 19 | L | 4 | 5 | 1.91 | US-guided biopsy | IDC Grade1 |
| 20 | L | 2 | 26 | 8.79 | US-guided biopsy | IDC Grade2 |
| 21 | L | 5 | 8 | 9.34 | US-guided biopsy | IDC Grade2 |
| 22 | L | 3 | 25 | 5.42 | US-guided biopsy | IDC Grade2 |
| 23 | L | 6 | 20 | 6.83 | US-guided biopsy | Mucinous carcinoma Grade1 |
| 24 | R | 3 | 20 | 6.70 | US-guided biopsy | IDC Grade2 |
| 25 | L | 2 | 13 | 8.49 | US-guided biopsy | IDC Grade3 |
| 26 | R | 3 | 10 | 7.32 | US-guided biopsy | Mucinous carcinoma Grade2 |
| 27 | R | 1 | 12 | 2.97 | US-guided biopsy | IDC Grade2 |
| 28 | L | 1 | 20 | 9.80 | US-guided biopsy | IDC Grade2 |
| 29 | L | 3 | 10 | 3.59 | US-guided biopsy | DCIS Grade2 |
| 30 | L | 10 | 22 | 6.42 | US-guided biopsy | IDC Grade3 |
| 31 | L | 2 | 13 | 7.33 | US-guided biopsy | ILC Grade2 |
| 32 | R | 11 | 17 | 3.32 | US-guided biopsy | IDC Grade2 |
| 33 | R | 11 | 34 | High (10.00) | US-guided biopsy | IDC Grade3 |
| 34 | R | 8 | 33 | 9.75 | US-guided biopsy | IDC Grade3 |
| 35 | R | 4 | 20 | 4.15 | US-guided biopsy | IDC Grade3 |
| 36 | L | 12 | 35 | 10.00 | US-guided biopsy | IDC Grade3 |
| 37 | R | 9 | 35 | 8.56 | US-guided biopsy | DCIS Grade2 |
| 38 | L | 12 | 9 | 4.61 | US-guided biopsy | IDC Grade2 |
| 39 | L | 5 | 12 | 6.48 | US-guided biopsy | IDC Grade2 |
| 40 | L | 12 | 13 | 8.98 | US-guided biopsy | IDC Grade2 |
| 41 | R | 10 | 54 | 4.74 | US-guided biopsy | IDC Grade3 |
| 42 | L | 2 | 16 | 5.64 | US-guided biopsy | IDC Grade1 |
| 43 | R | 10 | 20 | 2.68 | US-guided biopsy | Medullary carcinoma Grade3 |
| 44 | L | 11 | 18 | 5.00 | US-guided biopsy | ILC Grade2 |
| 45 | L | 12 | 22 | 9.23 | US-guided biopsy | DCIS Grade2 |
| 46 | L | 2 | 16 | 3.59 | US-guided biopsy | IDC Grade3 |
| 47 | L | 12 | 7 | 4.15 | US-guided biopsy | IDC Grade3 |
| 48 | R | 10 | 12 | 4.52 | US-guided biopsy | IDC Grade3 |
| 49 | L | 3 | 27 | 5.50 | US-guided biopsy | IDC Grade3 |
| 50 | L | 2 | 6 | 2.51 | US-guided biopsy | IDC Grade1 |
| 51 | L | 2 | 22 | 6.50 | US-guided biopsy | IDC Grade3 |
| 52 | R | 6 | 13 | 5.90 | US-guided biopsy | IDC Grade1 |
| 53 | L | 12 | 25 | 3.25 | US-guided biopsy | IDC Grade2 |
| 54 | R | 10 | 8 | 1.48 | US-guided biopsy | DCIS Grade1 |
| 55 | L | 4 | 18 | 6.43 | US-guided biopsy | IDC Grade2 |
| 56 | R | 9 | 20 | 9.18 | US-guided biopsy | IDC Grade3 |
| 57 | L | 12 | 10 | 7.16 | US-guided biopsy | IDC Grade1 |
| 58 | R | 12 | 35 | 9.86 | US-guided biopsy | IDC Grade3 |
| 59 | L | 3 | 37 | 9.99 | US-guided biopsy | IDC Grade2 |
| 60 | R | 10 | 19 | 8.10 | US-guided biopsy | IDC Grade2 |
| 61 | R | 5 | 40 | 2.43 | US-guided biopsy | DCIS Grade1 |
| 62 | L | 2 | 12 | 2.83 | US-guided biopsy | ILC Grade2 |
| 63 | L | 10 | 8 | 6.50 | US-guided biopsy | IDC Grade3 |
| 64 | L | 2 | 17 | 6.31 | US-guided biopsy | ILC Grade2 |
| 65 | L | 8 | 14 | 3.57 | US-guided biopsy | IDC Grade3 |
| 66 | L | 3 | 20 | 4.19 | US-guided biopsy | IDC Grade2 |
| 67 | L | 2 | 10 | 3.78 | US-guided biopsy | IDC Grade3 |
| 68 | L | 10 | 27 | 3.43 | US-guided biopsy | Angiosarcoma |
| 69 | L | 2 | 13 | 4.18 | US-guided biopsy | IDC Grade2 |
| 70 | R | 4 | 30 | 4.40 | US-guided biopsy | IDC Grade2 |
| 71 | L | 12 | 17 | 10.00 | US-guided biopsy | IDC Grade1 |
| 72 | L | 9 | 15 | 3.52 | US-guided biopsy | IDC Grade3 |
| 73 | R | 10 | 15 | 9.16 | US-guided biopsy | IDC Grade1 |
| 74 | R | 1 | 7 | 3.76 | US-guided biopsy | IDC Grade2 |
| 75 | R | 2 | 38 | 6.86 | US-guided biopsy | IDC Grade2 |
| 76 | R | 2 | 8 | 3.05 | US-guided biopsy | IDC Grade2 |
| 77 | R | 2 | 12 | 4.25 | Surgery | IDC Grade3 |
| 78 | R | 10 | 7 | 2.01 | Surgery | IDC Grade3 |
| 79 | L | 10 | 8 | 9.95 | Surgery | IDC Grade1 |
| 80 | L | 9 | 12 | 2.53 | Surgery | IDC Grade3 |
| 81 | L | 1 | 6 | 6.17 | Surgery | ILC Grade2 |
| 82 | L | 2 | 16 | 9.46 | Surgery | IDC Grade2 |
| 83 | L | 3 | 20 | 6.50 | Surgery | IDC Grade1 |
| 84 | L | 3 | 15 | 8.05 | Surgery | IDC Grade3 |
| 85 | R | 12 | 36 | 2.44 | US-guided biopsy | Fibrosis, fibroadenomatous hyperplasia |
| 86 | R | 7 | 8 | 2.06 | US-guided biopsy | Tumour-free parenchyma with microcalcifications |
| 87 | L | 10 | 10 | 2.44 | US-guided biopsy | Fibroadenoma |
| 88 | R | 2 | 5 | 1.81 | US-guided biopsy | Fat-necrosis |
| 89 | R | 1 | 8 | 3.20 | US-guided biopsy | Fibroadenomatous hyperplasia |
| 90 | L | 2 | 20 | 2.08 | US-guided biopsy | Galactocele with calcifications |
| 91 | R | 8 | 10 | 3.23 | US-guided biopsy | Apokrin metaplastic cyst, fat-necrosis |
| 92 | L | 1 | 31 | 3.73 | US-guided biopsy | Fibroadenoma |
| 93 | R | 8 | 23 | 2.13 | US-guided biopsy | Fibroadenoma |
| 94 | L | 9 | 9 | 2.06 | US-guided biopsy | Apocrine metaplasia |
| 95 | L | 12 | 20 | 3.66 | US-guided biopsy | Fibroadenomatous hyperplasia |
| 96 | L | 5 | 15 | 4.01 | US-guided biopsy | Fibroadenoma |
| 97 | R | 10 | 14 | 2.39 | US-guided biopsy | Cyst |
| 98 | L | 10 | 50 | 5.42 | US-guided biopsy | Granulomatous mastitis |
| 99 | L | 2 | 20 | 2.27 | US-guided biopsy | Pseudoangiomatous stromal hyperplasia |
| 100 | L | 5 | 20 | 2.75 | US-guided biopsy | Fibroadenoma |
| 101 | R | 1 | 12 | 2.48 | US-guided biopsy | Inflamed apocrine group of cysts |
| 102 | L | 9 | 10 | 2.49 | US-guided biopsy | Tumour-free parenchyma with secretory changes and calcifications |
| 103 | L | 3 | 11 | 5.23 | US-guided biopsy | Fibroadenoma |
| 104 | L | 2 | 40 | 6.50 | US-guided biopsy | Fibroadenoma |
| 105 | R | 8 | 7 | 3.61 | US-guided biopsy | Fibrosis |
| 106 | R | 9 | 15 | 1.46 | US-guided biopsy | Sclerosing adenosis |
| 107 | R | 12 | 20 | 3.21 | US-guided biopsy | Fibroadenoma |
| 108 | R | 1 | 7 | 1.99 | US-guided biopsy | Cyst |
| 109 | L | 3 | 15 | 6.50 | US-guided biopsy | Abscess |
| 110 | L | 2 | 25 | 2.24 | US-guided biopsy | Capillary Haemangioma |
| 111 | L | 2 | 10 | 1.42 | US-guided biopsy | Capillary Haemangioma |
| 112 | L | 2 | 9 | 2.89 | US-guided biopsy | Tumour-free hyalinised parenchyma |
| 113 | L | 7 | 7 | 2.79 | US-guided biopsy | Fibroadenomatous hyperplasia |
| 114 | L | 12 | 45 | 0.87 | US-guided biopsy | Abscess |
| 115 | L | 12 | 10 | 2.28 | US-guided biopsy | Fibroadenomatous hyperplasia |
| 116 | R | 3 | 17 | 3.14 | US-guided biopsy | Fibroadenoma |
| 117 | R | 12 | 10 | 2.62 | US-guided biopsy | Fibroadenoma |
| 118 | R | 7 | 8 | 1.44 | US-guided biopsy | Adenosis |
| 119 | R | 7 | 15 | 2.96 | US-guided biopsy | Fibrosis |
| 120 | R | 10 | 15 | 3.04 | US-guided biopsy | Papilloma |
| 121 | R | 9 | 10 | 4.23 | US-guided biopsy | Duct ectasia |
| 122 | L | 7 | 47 | 2.41 | US-guided biopsy | Apocrine metaplasia |
| 123 | R | 1 | 19 | 4.14 | US-guided biopsy | Fibroadenomatous hyperplasia |
| 124 | L | 12 | 35 | 7.15 | US-guided biopsy | Fibroadenoma |
| 125 | L | 2 | 22 | 2.81 | US-guided biopsy | Fibroadenoma |
| 126 | R | 7 | 11 | 2.59 | US-guided biopsy | Fibroadenomatous hyperplasia |
| 127 | R | 10 | 7 | 2.08 | US-guided biopsy | Inflammatory changes |
| 128 | R | 9 | 18 | 2.90 | US-guided biopsy | Fibroadenomatous hyperplasia |
| 129 | L | 3 | 15 | 2.36 | US-guided biopsy | Periductal mastitis |
| 130 | R | 3 | 8 | 1.18 | US-guided biopsy | Lipoma |
| 131 | R | 9 | 12 | 1.00 | US-guided biopsy | Tumour-free parenchyma |
| 132 | R | 9 | 5 | 2.31 | Surgery | Fat-necrosis |
| 133 | R | 9 | 14 | 3.57 | Surgery | Fat-necrosis |
| 134 | L | 12 | 29 | 2.95 | Surgery | Sclerosing adenosis |
| 135 | L | 4 | 6 | 2.09 | Surgery | Papilloma |
| 136 | L | 4 | 10 | 3.56 | Surgery | Papilloma |
| 137 | R | 9 | 5 | 1.86 | Surgery | Periductal mastitis |
| 138 | R | 10 | 35 | 4.08 | Surgery | Sclerosing/tubular adenosis and papillomas |
| 139 | L | 3 | 31 | 4.89 | Surgery | Fibroadenoma |
| 140 | L | 3 | 17 | 2.81 | Surgery | Papilloma |
| 141 | L | 2 | 11 | 0.98 | Follow-up stability |  |
| 142 | L | 7 | 18 | 2.51 | Follow-up stability |  |
| 143 | R | 10 | 13 | 2.07 | Follow-up stability |  |
| 144 | L | 3 | 25 | 1.71 | Follow-up stability |  |
| 145 | L | 2 | 25 | 9.76 | Follow-up stability |  |
| 146 | R | 3 | 13 | 2.04 | Follow-up stability |  |
| 147 | R | 4 | 36 | 9.37 | Follow-up stability |  |
| 148 | L | 2 | 9 | 3.17 | Follow-up stability |  |
| 149 | L | 10 | 8 | 2.53 | Follow-up stability |  |
| 150 | R | 1 | 7 | 2.72 | Follow-up stability |  |
| 151 | L | 9 | 12 | 2.56 | Follow-up stability |  |
| 152 | R | 9 | 7 | 2.41 | Follow-up stability |  |
| 153 | L | 2 | 9 | 1.82 | Follow-up stability |  |
| 154 | L | 12 | 16 | 3.38 | Follow-up stability |  |
| 155 | R | 1 | 10 | 2.39 | Follow-up stability |  |
| 156 | R | 12 | 12 | 3.64 | Follow-up stability |  |
| 157 | L | 11 | 13 | 2.73 | Follow-up stability |  |
| 158 | L | 12 | 13 | 2.11 | Follow-up stability |  |
| 159 | R | 6 | 10 | 2.07 | Follow-up stability |  |
| 160 | L | 2 | 12 | 1.03 | Follow-up stability |  |
| 161 | R | 2 | 7 | 3.35 | Follow-up stability |  |
| 162 | L | 3 | 14 | 1.70 | Follow-up stability |  |
| 163 | L | 2 | 15 | 2.34 | Follow-up stability |  |
| 164 | L | 3 | 13 | 1.18 | Follow-up stability |  |
| 165 | L | 3 | 10 | 2.98 | Follow-up stability |  |
| 166 | R | 1 | 20 | 4.81 | Follow-up stability |  |
| 167 | R | 9 | 11 | 2.01 | Follow-up stability |  |
| 168 | R | 7 | 5 | 1.58 | Follow-up stability |  |
| 169 | R | 10 | 43 | 2.83 | Follow-up stability |  |
| 170 | R | 10 | 8 | 3.15 | Follow-up stability |  |
| 171 | L | 11 | 5 | 1.95 | Follow-up stability |  |
| 172 | L | 9 | 15 | 1.85 | Follow-up stability |  |
| 173 | L | 2 | 12 | 1.92 | Follow-up stability |  |
| 174 | R | 10 | 14 | 3.67 | Follow-up stability |  |
| 175 | L | 12 | 18 | 4.21 | Follow-up stability |  |
| 176 | R | 10 | 6 | 1.97 | Follow-up stability |  |
| 177 | L | 4 | 6 | 2.20 | Follow-up stability |  |
| 178 | R | 4 | 8 | 6.12 | Follow-up stability |  |
| 179 | R | 10 | 30 | 2.86 | Follow-up stability |  |
| 180 | L | 1 | 6 | 1.66 | Follow-up stability |  |
| 181 | R | 7 | 11 | 2.52 | Follow-up stability |  |
| 182 | L | 6 | 7 | 2.87 | Follow-up stability |  |
| 183 | L | 2 | 21 | 9.79 | Follow-up stability |  |
| 184 | L | 2 | 20 | 4.25 | Follow-up stability |  |
| 185 | R | 9 | 16 | 1.87 | Follow-up stability |  |
| 186 | R | 10 | 22 | 2.78 | Follow-up stability |  |
| 187 | R | 11 | 13 | 1.50 | Follow-up stability |  |
| 188 | L | 11 | 8 | 2.51 | Follow-up stability |  |
| 189 | R | 11 | 12 | 2.67 | Follow-up stability |  |
| 190 | R | 12 | 15 | 2.69 | Follow-up stability |  |
| 191 | R | 1 | 19 | 2.07 | Follow-up stability |  |
| 192 | L | 9 | 10 | 1.45 | Follow-up stability |  |
| 193 | R | 2 | 6 | 1.75 | Follow-up stability |  |
| 194 | L | 6 | 35 | 2.08 | Follow-up stability |  |
| 195 | L | 2 | 22 | 2.63 | Follow-up stability |  |
| 196 | R | 9 | 6 | 1.35 | Follow-up stability |  |

Supplementary Table S1. Table containing all included lesions, with respective SWV and histology. SWV: Shear wave velocity, R: right L: left, US: ultrasound, IDC: invasive ductal cancer, ILC: invasive lobular cancer, DCIS: ductal carcinoma in situ.
